# Long Non-coding RNAs as Functional and Structural Chromatin Modulators in Acute Myeloid Leukemia

**DOI:** 10.3389/fonc.2019.00899

**Published:** 2019-09-11

**Authors:** Alexander A. Wurm, Cristina Pina

**Affiliations:** ^1^Department of Medical Translational Oncology, National Center for Tumor Diseases (NCT) Dresden, Dresden, Germany; ^2^Department of Genetics, University of Cambridge, Cambridge, United Kingdom

**Keywords:** acute myeloid leukemia, long non-coding RNA, chromatin regulation, epigenetic therapies, personalized medicine

## Abstract

Acute myeloid leukemia is a hematopoietic neoplasm of dismal prognosis that results from the accumulation of immature myeloid blasts in the bone marrow and the peripheral blood. It is strongly dependent on epigenetic regulation for disease onset, maintenance and in response to treatment. Epigenetic regulation refers to the multiple chemical modifications of DNA or DNA-associated proteins that alter chromatin structure and DNA accessibility in a heritable manner, without changing DNA sequence. Unlike sequence-specific transcription factors, epigenetic regulators do not necessarily bind DNA at consensus sequences, but still achieve reproducible target binding in a manner that is cell and maturation-type specific. A growing body of evidence indicates that epigenetic regulators rely, amongst other factors, on their interaction with untranslated RNA molecules for guidance to particular targets on DNA. Non (protein)-coding RNAs are the most abundant transcriptional products of the coding genome, and comprise several different classes of molecules with unique lengths, conformations and targets. Amongst these, long non-coding RNAs (lncRNAs) are species of 200 bp to >100 K bp in length, that recognize, and bind unique and largely uncharacterized DNA conformations. Some have been shown to bind epigenetic regulators, and thus constitute attractive candidates to mediate epigenetic target specificity. Herein, we postulate that lncRNAs are central players in the unique epigenetic programming of AML and review recent evidence in support of this view. We discuss the value of lncRNAs as putative diagnostic, prognostic and therapeutic targets in myeloid leukemias and indicate novel directions in this exciting research field.

## Foreword

The aim of this mini-review is to discuss emerging epigenetic roles of long non-coding RNAs (lncRNAs) in Acute Myeloid Leukemia (AML). AML has a characteristic dependency on chromatin and transcriptional regulators, which has been discussed in many reviews. To date, most of our understanding of epigenetic regulation and its participation in leukemogenesis is based on the enzymatic activities and protein-protein interactions of histone and DNA modifiers. However, a growing body of evidence suggests that recruitment, stability, and function of epigenetic factors can be mediated by non-protein-coding RNAs. In particular, there are incremental examples of the participation of the distinct class of long non-coding RNAs (lncRNAs) in AML epigenetic regulation, some of which have translational potential. In this context, we summarize common epigenetic alterations in AML that are relevant for understanding lncRNA potential contribution to the disease. We define lncRNAs and highlight their general roles of in normal development and in cancer. Finally, we review the developing body of literature on the participation of lncRNAs in epigenetic regulation of AML, and discuss their putative therapeutic relevance. We expect that the ideas advanced in this mini-review will be sedimented and extended in the coming years, thus positioning lncRNAs at the heart of epigenetic regulation and manipulation in AML.

## Epigenetic Alterations are Common Features of Acute Myeloid Leukemia

AML is an aggressive malignancy of the non-lymphoid lineages of the blood. It is a heterogeneous disease, cellularly and molecularly, with an overarching theme of ectopic self-renewal and arrested differentiation potential at multiple levels of the hematopoietic tree (1). Akin to the normal hematopoietic system, leukemias are sustained by a small number of leukemia stem-like cells (LSC), which can be distinct from the normal hematopoietic stem cells (HSC) but also exhibit functional characteristics of self-renewal and (abnormal or hindered) differentiation, and are often quiescent (2–4). Emergence of LSC is dependent on individual or combined genetic mutations that broadly determine the cellular affiliation of the leukemia, and permit or impose ectopic self-renewal and a restricted differentiation potential into what constitutes the proliferative bulk of the leukemia (5, 6). Different genetic lesions confer distinct natural histories, and hence prognosis. From a molecular point of view, AML has several distinctive features. It has a low mutation burden, particularly in the more extensively analyzed coding genome (7). A significant number of these mutations target transcription factors (TFs) and/or epigenetic regulators, thus indicating a specific reliance on gene regulatory mechanisms (8). The strong dependence of AML on epigenetic regulation has recently been highlighted by the prognostic value of heterogeneous DNA methylation epi-alleles: these are established *de novo* after chemotherapy and independently of mutation, and may drive disease progression and relapse in a manner akin to genetic variegation (9).

Epigenetic regulators act by modulating the accessibility of DNA to the basal gene expression machinery without absolute sequence-specificity, but with at least some degree of heritability across cell divisions (10). Broadly speaking, they can: (1) activate or repress gene expression through specific enzymatic activities that add or remove active residues from DNA or histones (e.g., acetyl, methyl, phosphor residues); (2) recognize specific modifications and recruit additional enzymatic activities or regulatory machinery to extend or erase the modification; (3) directly interact with general TFs and the RNA polymerases to initiate, maintain or terminate transcription. They can be classified as writers—active residue depositors, erasers—active residue removers, or readers—active residue binders, all classes having been implicated in pathogenesis of cancer in general, and of AML in particular (11).

## Epigenetic Alterations in AML Can Result From Mutations of Chromatin Regulators

Epigenetic regulators recurrently mutated in AML include: Histone acetyl-transferases (HATs)—namely *EP300/CBP* and members of the *MYST/MOZ* family, through point mutation or chromosomal translocation (12). Histone methyl-transferases—namely *KMT2B/MLL1*, a chromosomal translocation hotspot leading to a uniquely aggressive disease of bad prognosis (13). DNA methyl-transferases and demethylases—respectively *DNMT3A* (14, 15), and *TET2* (16, 17) or the metabolic enzymes *IDH1/2* (18, 19), that interfere with target-specific methylation, and can also reorganize DNA methylation patterns genome-wide (20, 21). Point mutations in *DNMT3A* have mutation-specific effects on DNA methylation (22). Point mutations in *TET2* interfere with its capacity to demethylate DNA, with repressive consequences for gene expression (23). These are broadly mimicked by gain-of-function point mutations in the metabolic enzymes *IDH1* and *IDH2*, which inhibit TET activity through accumulation of the methylation intermediate 5-hydroxy-methyl cytosine (5-hmC) (24). Mutations in the Cohesin complex perturb higher-order genome organization (25). Other commonly mutated genes affect gene expression regulators downstream of transcription, namely splicing and assembly of the translational machinery (8). In the latter group, the nucleus-to-cytoplasm pre-ribosomal transporter *NPM1* is the most commonly targeted gene in AML (26).

## Leukemogenic Programs Co-opt Non-mutated Epigenetic Regulators

Importantly, AML cells are also strictly dependent on a number of non-mutated epigenetic regulators that are co-opted by the oncogenic programs to maintain self-renewal and/or survival of the leukemic cells. Some of these epigenetic regulators are central to the leukemic program in specific mutation contexts. The H3K79me2/3 methyl-transferase DOT1L is recruited by several of the MLL fusion genes, e.g., *MLLT1/ENL* or *MLLT3/AF9*, as part of the RNA polymerase (Pol) II super-elongation complex to modify promoters and proximal gene bodies and activate transcription (27). Histone deacetylases (HDACs) are recruited by leukemic fusions involving the master regulator *RUNX1* gene or its binding partner *CBFB* (in the so-called core-binding factor AMLs) to repress transcription at specific targets (28–30). Other epigenetic regulators are recruited more broadly across the AML mutational spectrum: the Bromodomain protein 4 (BRD4), a reader of lysine acetylation, was originally identified as a SEC participant in the context of MLL fusion AML (31), but has since been shown to exert a more general influence in maintaining AML gene expression programs, including in the frequent genetic contexts of *NPM1* mutation (32), and of the oncogenic Fms-like Tyrosine Kinase 3 internal tandem duplication (*FLT3-ITD*) (33) that constitutively activates signaling from the hematopoietic growth factor receptor FLT3 (34). *LSD1*, or *KDM1A*, is a histone demethylase that removes mono and di-methylation from H3K4 and H3K9, with repressive or activating effects on gene expression (35, 36). Like BRD4, it was initially described as a requirement in *MLL* re-arranged AML (37), but has more recently been shown to inhibit AML cell differentiation more generally (38). We have added the histone acetyl-transferase KAT2A to the list of putative global requirements in AML. KAT2A is primarily required for acetylation of H3K9 in gene promoters (39), but it can also acetylate non-histone proteins, including transcription factors EGR2 (40) and C/EBPα (41), which are relevant for normal and malignant hematopoiesis. We identified KAT2A as a genetic vulnerability in a subset of AML cell lines in a genome-wide CRISPR drop-out screen, and showed a requirement for self-renewal and/or survival in patient samples across a spectrum of mutational events (42). It also a requirement in ES cells (43, 44) and induced pluripotent stem cells (45), and its interaction with the MYC oncogene may translate into AML. Recently, the methyl-transferase EZH2, a member of the Polycomb Repressive Complex 2 (PRC2) that deposits the repressive H3K27me3 modification, has been characterized as both a tumor suppressor and an oncogene in AML, depending on the stage of the disease (46). Specifically, loss of EZH2 promotes AML initiation, reflected in low-frequency loss-of-function mutations in myeloid neoplasms (47–49), and the observed acceleration of AML transformation from pre-malignant myeloproliferative neoplasms (MPN) (50, 51). On the other hand, established AML (52, 53), as well as chronic myeloid leukemia (CML) cells (54), depend on expression of EZH2 for disease maintenance. and there is potential for EZH2 inhibitors as a therapeutic strategy in established AML.

In summary, AML depends on multiple epigenetic regulators, in genetic abnormality, disease subtype, and disease stage-dependent manners, with and without mutation of the epigenetic regulators themselves. Unsurprisingly, this is an area of intensive research that holds promise in the development of novel anti-leukemia therapeutic strategies.

## Long Non-coding RNAs are Largely Unexplored Transcriptional Species

It is known that only ~3% of the human transcriptome is translated into functional proteins (55). Thus, the vast majority of transcribed genes remain at the RNA level. The largest part of this group comprises ribosomal RNAs (rRNAs) and transfer RNAs (tRNAs). The remaining fraction is referred to as “non-coding RNAs” (nc-RNAs) and can be stratified into distinct families, mainly according to the RNA length. There are small nc-RNAs, namely microRNAs, piwi-interacting RNAs or sno-RNAs, which range from below 40 to ~200–300 bp in length. At the other end of the spectrum, lncRNAs are a heterogeneous group that exhibit a wide size range from at least 200 bp and up to 100 kbp (56). There have been inconsistent attempts at lncRNAs classification, with tentative subdivisions on the basis of genomic localization or molecular function (57). As a potential confounder, several studies revealed strong structural, but not sequence, conservation of lncRNAs between different organisms (58), indicating that inter-species comparison such as used for protein-coding genes may not systematically predict the biological relevance of lncRNAs. LncRNAs have been associated with a plethora of regulatory functions, including regulation of gene expression and alternative splicing, DNA-RNA-protein interactions, and participation in histone-DNA complexes (59). A breakthrough observation in mouse embryonic development positioned lncRNAs as epigenetic regulators. Specifically, the lncRNA *HOX transcript antisense intergenic RNA (HOTAIR*), which is transcribed from the *HOXC* locus in a tissue-specific manner, was found to mediate transcriptional silencing of the *HOXD* gene locus through direct interaction with the PRC2. This provided the first evidence that non-coding RNAs could regulate epigenetic gene silencing. Subsequently, various other lncRNAs, e.g., *Xist, RepA, Kcnq1ot1, Braveheart*, or *Malat-1*, have been shown to interact with PRC2 in a multi-protein-RNA complex (60). Indeed, on the basis of global RNA immunoprecipitation (RIP) experiments, ~20% of human lncRNAs interact with PRC2 (61), highlighting the influence of the long non-coding RNAome on transcriptional activity.

## LncRNAs Can Act as Epigenetic Drivers in Human Cancer

In contrast with other ncRNA species, e.g., microRNAs, which have been intensively studied in cancer, including leukemia (76, 77), lncRNAs have only recently been recognized as active players in initiation, maintenance and treatment response of human cancer (78).

In some instances, lncRNA contribution to tumor biology results from direct epigenetic regulation or participation in chromatin complexes. LncRNAs are actively transcribed by RNA-PolII (in some instances, RNA-PolIII), and their expression can be modulated by chromatin-modifying complexes. For example, the lncRNA *Low Expression in Tumor (LET)* is transcriptionally silenced in various solid cancers by H*istone Deacetylase 3 (HDAC3)*-mediated promoter inactivation (79). Also, the lncRNA *Urothelial Cancer Associated 1 (UCA1)* is regulated by ARID1A, a component of the SWI/SNF chromatin-remodeling complex (80), and itself interacts with multiple histone-modifying proteins to regulate target gene expression (81).

More importantly, lncRNAs can contribute to cancer by epigenetic activation or silencing of target loci in a cis or trans-regulatory manner. LncRNAs can modify gene expression in *cis* by altering chromatin structure or DNA methylation pattern of neighboring gene promoters. Approximately 40% of all human protein coding genes are co-expressed with a paired natural antisense transcript (NAT), a lncRNA subtype that is expressed from the opposite strand of the host gene (82). Host gene-NAT ratios are often tissue-dependent and have been shown to clearly define cancer subtypes (82). Antisense lncRNAs can protect paired host genes from epigenetic silencing by interacting with histone-modifying enzymes such as the PRC2 (60). In contrast, host gene-associated antisense RNAs can also silence paired protein-coding genes, as exemplified by the acetylcholinesterase *(AChE)* gene, which is silenced by *AChE-AS* lncRNA in hepatocellular carcinoma through triggering of histone methylation (83). Interactions between gene and antisense-RNA can impact disease progression, as in the increased chemotherapy resistance associated with transcriptional activation of *GAS6-AS2* lncRNA in multiple cancers including AML (84). In addition to anti-sense pairing, lncRNAs can also exert *cis*-regulatory activity when transcribed in sense with a host gene. Exemplarily, the *diRNA* (DNMT1-interacting RNAs) lncRNA family modifies global DNA methylation patterns by protecting host gene promoters from DNMT1-driven *de novo* methylation after cell division (75).

There are several examples of *trans*-regulatory activities of lncRNAs that modify gene expression through chromatin remodeling. In gastric cancer, *GClnc1* regulates a plethora of target genes by recruiting histone-modifying proteins KAT2A and WDR5 and activating gene expression (85). As discussed, KAT2A is a HAT with novel putative roles in AML maintenance. WDR5, itself an RNA binding protein, is an essential subunit of the MLL complex that drives H3K4me3 (86). WDR5 is also a component of the Ada-2-a-containing (ATAC) complex, one of the 2 complexes through which KAT2A exerts its HAT activity, suggesting an additional level of putative interaction between *GClnc1* and the two histone modifiers in promoting gene expression. LncRNA-mediated trans-regulation of gene loci can equally lead to gene repression. Various lncRNAs including *Xist, TUG1*, and *HOTAIR* show the potential to interact with PRC2 and mediate target gene promoter inactivation in cancer (87–89). For instance, the p53 target lncRNA *Taurine-Upregulated Gene 1 (TUG1)* is dramatically downregulated in lung cancer. Mechanistically, it can silence the *HOXB7* oncogene expression by recruiting EZH2 to the core promoter and repressing gene expression through deposition of H3K27me3 (89). In summary, lncRNAs are both mediators and targets of deregulated epigenetic cascades in human cancer, and can contribute to cancer initiation and progression at multiple levels.

## Histone Modification in AML is Guided by LncRNAs

Knowledge about lncRNA function in AML onset, progression and response to treatment is in its infancy. LncRNAs have been shown to maintain or enhance LSC self-renewal, as exemplified by the dramatic reduction in the number of active LSC in an AML mouse model upon knockdown of lncRNA *DANCR* (90). They can promote leukemogenesis by maintaining active transcription, as described for *LINC00152*, which protects the elongating PolII subunit CDK9 from miR-193a induced silencing through a miRNA sponge-like effect (91). Globally, defined lncRNA expression patterns in AML bone marrow or peripheral blood samples exhibit strong correlation with clinical outcome and are suitable for AML subtype stratification (92–94). Important from a clinical perspective, individual lncRNA quantified in serum samples also have prognostic value and can be used as liquid biopsy-based biomarkers. For example, high serum levels of *LINC00899* associate with reduced overall survival and can clearly discriminate between AML patients and healthy controls (95). Despite the clinical relevance of individual lncRNAs, to date no sequence-specific mutations in their genes (single nucleotide variants, SNV, insertions or deletions) have been described in AML. The same is true of another hematological malignancy, chronic lymphocytic leukemia (CLL), in which analysis of the non-coding genomic landscape of patients failed to identify recurrent mutations in lncRNA genes (96). This is in contrast with solid cancers, where various clinically and biologically relevant SNPs in lncRNA genes have been described (97). It is possible that lncRNA mutations are rare in leukemias and do not often contribute as leukemia drivers. Rather, lncRNAs may mediate malignant transformation events in hematopoietic cells through deregulated expression. Published examples are summarized in Table 1, and we will discuss them here in more detail. They illustrate similar regulatory mechanisms to those described in solid cancers, namely epigenetic deregulation of lncRNA expression and interaction with the chromatin-modifying machinery, as well as lncRNA-mediated cis and trans-regulation of individual loci.

**Table 1:** Overview about lncRNA involved in epigenetic reprogramming in AML.

**lncRNA**	**Regulator**	**Target**	**Function**	**References**
*Xist*	RNF12	PRC2	Suppressor of AML/MDS development	(62, 63)
*MEG3*	WT1	TP53	Suppressor of AML development in a p53 dependent and p53 independent manner	(64)
*LINC01268*	*HDAC2*	*HDAC2*	Associates with poor prognosis in AML and increases proliferation. Positive feedback loop with *HDAC2*	(65)
*HOTAIR*	NF-kB, BRD4, MYC	PRC2 LSD1/CoREST/REST	Associates with poor prognosis in AML, inhibits p15, induces self-renewal in leukemia initiating cells	(66–71)
*HOTTIP*	HOX genes	WDR5-MLL	Induces AML like phenotype in mice	(72, 73)
*UCA1*	C/EBPα-p30	hnRNP I, p27^kip1^	Induces proliferation by repression of p27^kip1^	(74)
*ecCEBPA*	C/EBPα	DNMT1	Protects CEBPA gene locus from methylation in AML cell lines	(75)

In the first category of epigenetic deregulation, it has been observed that AML-associated lncRNA genes have overall low DNA methylation and high H3K4me3 compared to healthy hematopoietic stem and progenitor cells (65). This results in higher lncRNA expression levels in AML and illustrates putative oncogene-type roles. Other lncRNAs can act as tumor suppressors, as shown for *X-inactive specific transcript (Xist)*, the lncRNA central to X-chromosome inactivation (62): its depletion in HSCs and progenitors leads to the development of a myeloproliferative disorder through interaction with PRC2 (87). Also, epigenetic silencing or inactivating mutations of the *Wilms' Tumor 1 (WT1)* gene result in repression of *Maternally Expressed Gene 3 (MEG3)* lncRNA expression, a tumor suppressive lncRNA that inhibits tumor growth in a TP53 dependent and independent manner. Inactivation of *MEG3* in turn promotes AML initiation (64). These examples illustrate the importance of epigenetic changes in the establishment of enhanced or aberrant lncRNA expression.

**Figure 1:**
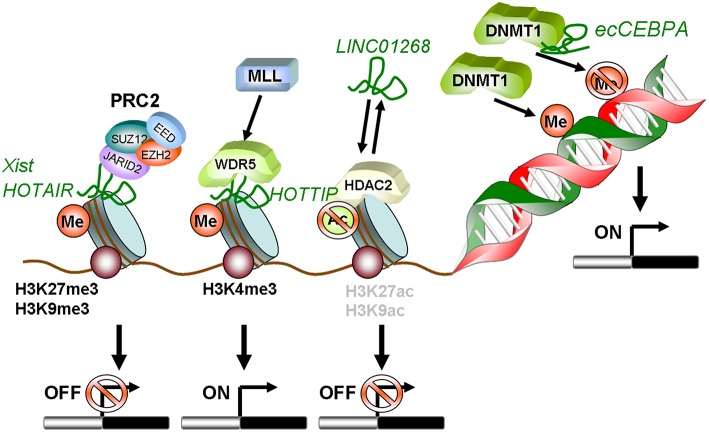
Schematic summary of lncRNAs involved in epigenetic regulation in AML. *HOTAIR* and *Xist* can recruit PRC2 to methylate histones on target gene promoters leading to transcriptional silencing. *LINC01268* activates *HDAC2*, which in turn deacetylates H3 on K9 and K27. This also induces transcriptional silencing. In contrast, lncRNA *HOTTIP* guides WDR5 to H3K4 and recruits the MLL complex. This mediates tri-methylation and activation of transcription. The diRNA familiy member *ecCEBPA* directly interacts with DNMT1 and functions as a shield to protect the *CEBPA* promoter from DNA methylation to ensure constant expression.

Locus regulation and downstream interaction with chromatin modifiers further illustrate the epigenetic role of lncRNAs in AML. For example, lncRNA *LINC01268*, located ~60 kb upstream of the *HDAC2* gene, directly activates *HDAC2* expression in a *cis*-regulatory manner. In turn *HDAC2* triggers *LINC01268* transcription to establish a positive feedback loop (65). One of the most prominent examples involves lncRNA *HOTAIR*, which associates with poor prognosis in AML (66). Mechanistically, *HOTAIR* binds simultaneously to PRC2 and to the LSD1/CoREST/REST complex and acts as a chromatin scaffold that couples PRC2-mediated repressive trimethylation of H3K27 with demethylation of H3K4 and the consequent inactivation of the specific loci (67). In AML, *HOTAIR* ensures the stem cell-like behavior of LSCs by mediating EZH2-driven H3K27me3 of the *CDKN2B*/*P15-INK4b* promoter. *CDKN2B* encodes for the P15 cyclin-dependent kinase inhibitor, and reducing its expression promotes proliferation and self-renewal (68). Maintenance of AML self-renewal is also achieved through induction of the lncRNA *urothelial carcinoma associated 1* (*UCA-1*) in C/EBPα mutant AML. C/EBPα is a master transcription factor regulator of myeloid cell differentiation, which is recurrently mutated in AML. The mutant truncated p30-isoform of C/EBPα promotes *UCA-1* expression, which in turns promotes leukemia proliferation through recruitment of the non-coding RNA-protein complex hnRNP I to repress the cyclin-dependent kinase inhibitor P27Kip1(74). *HOXA distal transcript antisense RNA (HOTTIP)* is a lncRNA located within the *HOXA* gene cluster (72). *HOXA7-11* genes are central to AML self-renewal programs, namely in the context of *MLL* rearrangements and *NPM1* mutation. Within the locus, *HOTTIP* activates expression of *HOX* genes in a candidate positive feedback loop: it guides the WDR5-MLL methyl-transferase complex to the respective promoters, resulting in deposition of the activating H3K4me3 modification (72). Accordingly, in a hematopoiesis-specific transgenic mouse model, increased *HOTTIP* expression leads to increased WBC and neutrophil counts, to a splenomegaly, and an altered self-renewal—differentiation balance of hematopoietic stem and progenitor cells (73). In summary, lncRNAs may contribute to AML initiation and maintenance through close regulatory interaction with the epigenetic machinery. Figure 1 illustrates these regulatory modes, which will continue to expand in coming years.

## Hypothesis, Conclusions, and Perspectives

The field of lncRNAs in cancer, including AML, is in its infancy. However, a growing body of data provides strong evidence for the role of lncRNAs as key mediators and effectors of epigenetic alterations in AML, which will add to more advanced research in the epigenetic field. Indeed, several epigenetic-modifying drugs have either been approved for AML treatment, or are in clinical trials with initial promising results (98). This notwithstanding, first-line therapy remains a combination of cytotoxic agents cytarabine and an anthracycline, which have an associated toxicity often not tolerated by the elderly patients more commonly affected by the disease. Newly-introduced chemicals offer therapeutic alternatives to frailer patients and have improved rates of remission, but have not yet improved survival. Detailed understanding of epigenetic and other regulatory mechanisms, and the design of individualized therapeutic regimens, are thus paramount in AML. LncRNAs emerge as promising new players in this task. This is illustrated in the case of DNA demethylating agents 5-azacytidine and decitabine, which have shown promising results in AML and related myelodysplastic syndromes (MDS) (99), but for which the molecular mechanisms are not fully uncovered and a significant proportion of patients fail to respond or acquire resistance (100). Although both agents show a non-random reproducible demethylation pattern in cell lines (101) they do not exclusively affect tumor-specific programs and might produce undesired side effects. Recently, diRuscio et al. unveiled a mechanism by which cells can use lncRNAs endogenously for target-specific demethylation (75), with direct potential therapeutic implications. In the example studied in AML cell lines, the *ecCEBPA* lncRNA directly binds to the DNMT1 protein and protects the *CEBPA* gene promoter from DNMT1-mediated *de novo* methylation after cell division. This mechanism tightly controls the transcriptional activity of the *CEBPA* host gene with promoter specificity; furthermore, it suggests a template for future targeted cancer therapies using lncRNAs as scaffolds to control demethylation of individual hypermethylated targets. In theory, this strategy can increase efficiency and decrease toxicity of untargeted DNA demethylating agents. In addition to demethylating therapies, HDAC inhibitors have also been developed as potential epigenetic therapeutic agents (102). However, their success remains limited, with challenges arising from poor drug selectivity, toxic side effects and lack of informative biomarkers to predict and guide selection of responding patients (103). LncRNAs have been proposed to regulate the balance between histone acetylation and deacetylation activities (104), and, similarly to specification of demethylation activities, they may potentially be used as molecular scaffolds to trigger target promoter/enhancer-specific chromatin acetylation, with fine and directed control of AML-relevant locus transcription.

In conclusion, preliminary data indicate strong potential for lncRNAs as biomarkers, prognostic factors, and therapeutic targets or mediators in AML, with the ability to individualize therapies, reduce toxicity and enhance treatment success, all of them urgent roles in this disease.

## Author Contributions

All authors listed have made a substantial, direct and intellectual contribution to the work, and approved it for publication.

### Conflict of Interest Statement

The authors declare that the research was conducted in the absence of any commercial or financial relationships that could be construed as a potential conflict of interest.
